# Effects of spatial dimensionality and steric interactions on microtubule-motor self-organization

**DOI:** 10.1088/1478-3975/ab0fb1

**Published:** 2019-04-23

**Authors:** Jamie Rickman, François Nédélec, Thomas Surrey

**Affiliations:** 1 The Francis Crick Institute, 1 Midland Road, London NW1 1AT, United Kingdom; 2 Centre for Mathematics and Physics in the Life Sciences and Experimental Biology, University College London, London WC1 6BT, United Kingdom; 3 European Molecular Biology Laboratory, Meyerhofstrasse 1, 69117 Heidelberg, Germany; nedelec@embl.de; thomas.surrey@crick.ac.uk

**Keywords:** active networks, self-organisation, microtubules, molecular motors, computer simulations, Cytosim

## Abstract

Active networks composed of filaments and motor proteins can self-organize into a variety of architectures. Computer simulations in two or three spatial dimensions and including or omitting steric interactions between filaments can be used to model active networks. Here we examine how these modelling choices affect the state space of network self-organization. We compare the networks generated by different models of a system of dynamic microtubules and microtubule-crosslinking motors. We find that a thin 3D model that includes steric interactions between filaments is the most versatile, capturing a variety of network states observed in recent experiments. In contrast, 2D models either with or without steric interactions which prohibit microtubule crossings can produce some, but not all, observed network states. Our results provide guidelines for the most appropriate choice of model for the study of different network types and elucidate mechanisms of active network organization.

## Introduction

Active networks of filaments and molecular motors constitute the core of the cytoskeleton in eukaryotic cells and self-organize into a variety of distinct architectures which are critical for biological function. Experiments with purified proteins or cell extract have played an important role in elucidating the design principles of the cytoskeleton [1–3]. They have allowed for the quantitative characterization of active network self-organization in a simplified and highly tunable setting. Because these systems fall outside the remit of classical equilibrium thermodynamics [4], new theories of active matter have been developed alongside these experiments [5–7]. We focus here on active networks of microtubules and microtubule-crosslinking motors, which can generate a variety of network architectures from a small set of well-characterized components.

For example, locally contractile radial microtubule arrays, known as asters, have been generated from dilute mixtures of motors and microtubules [8–10]. The ability of motors to accumulate at microtubule ends where they form the foci of contraction is thought to be critical for the organization of this network type [11]. Tuning the concentrations of motors and microtubules can change the network behavior from local contraction and aster formation to global contraction of the entire network [12–14].

In contrast, higher microtubule densities can lead to the generation of aligned microtubule bundles which form ‘active nematic’ networks [15]. Microtubule alignment has been promoted by crowding agents producing a depletion force between microtubules [16, 17]. In the absence of crowding agents active nematic networks can also form at sufficiently high microtubule concentrations and growth speeds [13]. In these networks, bundles of aligned microtubules extend as motors slide anti-parallel microtubules apart, resulting in large-scale motion known as active turbulence [18]. When confined to a surface an active nematic network can display the topological defects characteristic of 2D liquid crystals [19], but in active nematics these defects are motile [20, 21].

Continuum theories of microtubule-motor networks based on coarse-grained filament orientation and velocity fields have successfully captured a number of the observed network states [22–24] and qualitatively describe the dynamics of defect motility [18, 25, 26]. These approaches use constitutive equations to calculate macroscopic quantities, typically assuming phenomenological parameters that do not map directly onto the measurable, microscopic properties of the molecular components. On the other hand, these properties can often be implemented in a detailed computational model in which filaments and motors are explicitly represented [9, 10, 27–36]. However, this complementary approach is typically limited to small system sizes due to the associated computational costs.

Most microscopic models have been built on the idea that motors exert forces by their motion which move filaments in a viscous, Brownian medium. But the level of complexity and the details of implementation have differed considerably between models. The most striking differences appear to arise from the choice of spatial dimensionality and the inclusion/exclusion of direct filament-filament interactions representing repulsive steric or attractive depletion effects. These important decisions are often guided by intuition or convenience, without knowing how these choices will affect the range of network architectures that a simulated system can represent.

Two-dimensional models without steric interactions are the simplest models. They are sufficient to recapitulate aster formation [10, 11], but not nematic states [37]. When steric interactions have been included in 2D models, aster states have been confined to a low-density regime [31], while nematic states [29, 31, 32, 36, 38] and polar-aligned networks [39, 40] have been well-represented.

However, the presence of steric interactions does not preclude the possibility of contractile states; isotropic global contractions have been observed in a 3D model of volume-excluding filaments [28] and locally contractile asters have been reproduced in thin 3D volumes [13, 41].

A systematic evaluation of the effects of spatial dimensionality and steric interactions on the outcome of microtubule-motor self-organization is currently missing. Here, we have compared the results from three different models of the same microtubule-motor system which differ with respect to the dimensionality of the simulation space (two or thin three-dimensional) and the inclusion/exclusion of steric interactions between filaments which prohibit their crossing in the plane. We examined how steric effects and dimensionality affected the formation of three archetypal microtubule-motor network states; active nematic networks, locally contractile asters and globally contractile networks. By controlling the ability of microtubules to cross, we found that steric forces and spatial dimensionality play critical and distinct roles in each network state and influence the state space of microtubule-motor self-organization represented by a given model. We finally make recommendations about appropriate modelling choices.

## Methods

### Model

**Figure 1.**
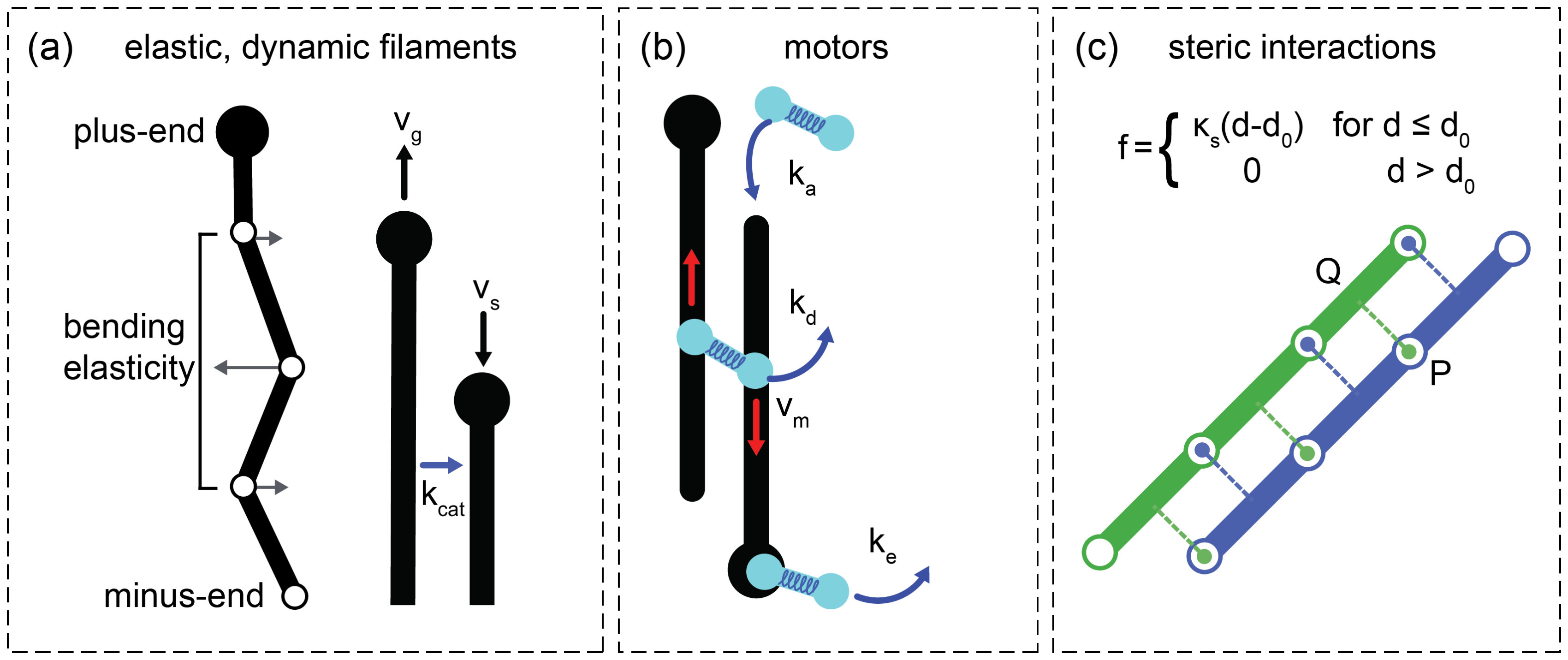
Essential elements of Cytosim. (a) Microtubules exhibit two-state dynamic instability [43] with growth rate ${{v}_{g}}$, shrinkage rate ${{v}_{s}}$ and catastrophe rate ${{k}_{cat}}$. They are discretized into points connected by inextensible segments such that microtubules can bend but cannot stretch. Points are subjected to forces from bending elasticity, steric interactions (if present) and crosslinking motors. (b) Motors consist of two motor entities. Each motor entity can bind, unbind and move along a microtubule. Crosslinking motors exert forces on the microtubules they connect via a Hookean spring-like link. (c) Steric interactions are calculated for each model point of a microtubule. For example, a line from *P*, a point on the blue microtubule, is projected onto the nearby segment of the green microtubule at *Q*. The line *PQ* is orthogonal to the green microtubule. An equal and opposite force is applied to the green and blue microtubule along *PQ* such that the steric forces acting on a pair of microtubules are symmetric and sum to zero.

The model was implemented in the open-source cytoskeletal simulation software Cytosim. The elements of our model are dynamic microtubule filaments, steric interactions between filaments and motors which walk to microtubule plus-ends (figure 1). Microtubules are discretized into a set of points, which form an inextensible backbone of infinitesimal width. The points of the microtubules are subject to bending elasticity, forces from crosslinking motors and steric interactions if present. The motion of all the points in the system is then calculated with a multi-dimensional over-damped Langevin equation, modelling Brownian dynamics in a viscous fluid as previously described [42].

Our model includes microtubule dynamics and maintains a steady number of microtubules in the presence of polymerization/depolymerization turnover. Initially, a fixed number of randomly distributed microtubule ‘nucleators’ create microtubules at rate ${{k}_{nuc}}$ with a short initial length ${{L}_{0}}$. The microtubule minus-ends (attached to the nucleator) are static but their plus-ends undergo dynamic instability. These conditions mimic self-organization assays that have been shown to generate a wide variety of network states [13]. Dynamic instability is implemented with a standard two-state model without rescue parameterized by a growth speed ${{v}_{g}}$, a catastrophe frequency ${{f}_{cat}}$ and a shrinkage speed ${{v}_{s}}$ [43]. A shrinking microtubule vanishes once it is shorter than a minimum length ${{L}_{min}}$ and its nucleator is then free to nucleate again. The ratio of motor speed to microtubule growth speed was recently shown to be a control parameter in the organization of extensile nematic networks and polar contractile networks [13]. In order to explore these archetypal microtubule-motor network states we therefore decided to tune this parameter by holding motor speed constant while varying the microtubule plus-end growth speed.

For the simulation of active nematic networks and asters, the growth speed, shrinkage speed and catastrophe frequency are constant throughout the simulation. An alternative growth condition, a ‘fast initial growth regime’, was implemented to explore the globally contractile state in which the microtubule growth speed is set dynamically from the total length of the microtubules at a given time point i.e. ${{v}_{g}}\left(t \right)=\alpha \left(1-\frac{1}{\Omega }\sum{{{L}_{i}}\left(t \right)} \right)$ where $\alpha $ is the maximum growth speed, $\sum{{{L}_{i}}\left(t \right)}$ is the total length of microtubules at time $t$ and the constant $ \Omega $ represents an upper bound on the total microtubule length. These assumptions intend to represent conditions in which the amount of tubulin from which microtubules polymerize is finite. In this ‘fast initial growth’ regime the growth speed is initially fast when the microtubules are newly nucleated and plateaus as the total microtubule length approaches its steady-state value. The speed $\alpha $ and the length $ \Omega $ were chosen so that ${{v}_{g}}$ in the steady-state was the same under both growth conditions. Shrinkage speeds were fast, such that the population of shrinking microtubules was small relative to the population of growing microtubules and shrinking microtubules thus had an insignificant effect on network organization.

Motors are modelled as a pair of motor entities connected by a Hookean spring-like link with resting length ${{d}_{m}}$ and stiffness ${{\kappa }_{m}}$. Each motor entity can bind and move along a microtubule. Motors with both motor entities bound to a microtubule crosslink them and exert force between them. The spring-like link can rotate freely at both attachment points such that the angle between two crosslinked microtubules is unconstrained. Unbound motors are treated as point-like particles. However, the diffusion of unbound motors is not modelled explicitly; it is assumed to be sufficiently fast that a uniform spatial distribution of unbound motors is maintained. Essentially, only a count of the number of unbound motors is kept and at each time step a fraction of these motors is directly attached by one (randomly chosen) motor entity at random positions along the filaments, while the other motor entity is kept unbound. An unbound motor entity can bind to any microtubule within a range $\varepsilon $ at rate ${{k}_{a}}$. Whereas motor binding and unbinding are stochastic events, bound motors move deterministically towards the plus-end of the microtubule at a speed which depends on its load vector ***f* ** as ${{v}_{m}}={{v}_{0}}\left(1+\boldsymbol{f}\cdot \boldsymbol{d}/{{f}_{s}} \right)$, where ***d*** is a unit vector in the direction of the microtubule, ${{f}_{s}}&gt;0$ is a characteristic stall force and ${{v}_{0}}&gt;0$ is the unloaded speed of the motor. Antagonistic forces are antiparallel to ***d*** and reduce motor speed. Motors detach from the microtubule lattice at a rate ${{k}_{d}}$. If they reach the microtubule end they do not unbind instantaneously but can dwell there and detach at rate ${{k}_{e}}$. The ability of motors to dwell at microtubule ends has been shown theoretically to be important in aster formation [10]. Moreover, both detachment rates are modulated exponentially by the load on the motor and a characteristic unbinding force ${{f}_{u}}$, according to Kramer’s law: ${{k}_{\left[ d,e \right]}}exp\left(\left\Vert \boldsymbol{f} \right\Vert/{{f}_{u}} \right)$.

To study the effects of steric interactions and dimensionality on the self-organization of the system, we compared three different models. Two of the models are 2D with square, periodic boundaries; the first model allows microtubules to cross in the absence of steric interactions (2D), while the other includes steric interactions between filaments which penalize microtubule crossings (2D  +  S). The *X*- and *Y*-dimensions of the simulation space were chosen to be large enough to mimic an unbounded region; the dimensions were 16*L*  ×  16*L*, with *L* (5 *μ*m) the average microtubule length. The third model is 3D in space and includes steric interactions (3D  +  S). The simulation space is confined and thin in the *Z*-direction and periodic in the *X* and *Y* directions. The confining force is derived from a harmonic potential that is flat inside the simulation space and rises quadratically away from its edges. Confining forces acting on microtubule points are orthogonal to the *X*-*Y* plane, such that the edges are frictionless. With the confinement in *Z* being smaller than *L* (*Z*  ≈  0.04*L*), microtubules tended to align in the *X*-*Y* plane but can pass each other in the third dimension, allowing apparent ‘crossings’ to be formed if the system is projected onto the *X*-*Y* plane. The *Z*-dimension of the 3D  +  S model was chosen such that three microtubules could overlap in the *X*-*Y* plane without interacting sterically with one another.

Steric interactions between filaments can be implemented in a number of ways [33, 34]. Here we chose a straightforward and widely used implementation that is expected to give realistic results in our simulations. To set the steric interactions between microtubules, the points and segments of every microtubule are tested against the segments of nearby microtubules. A projection from each point is made orthogonal to the nearby segments (figure 1(c)). If the projected distance is smaller than a specified threshold, an equal and opposite force is applied along this projection to the microtubule point and the nearby microtubule segment (the force applied to the nearby segment is distributed between the points enclosing the segment). The force is orthogonal to the nearby segment to minimize friction between microtubules, since steric interactions should not normally prevent sliding of filaments. If two filaments are straight and parallel, the steric forces between them are orthogonal to both filaments (figure 1(c)). In three dimensions an additional procedure is carried out to identify microtubule crossings in the third dimension that are not detected by the procedure described above; segments of nearby microtubules are checked in pairs and if an intersection is found a steric interaction is set along the path of the shortest distance between the segments (orthogonal to both segments).

The steric force is described by Hookean soft-core repulsion; $\left\Vert \boldsymbol{\,f} \right\Vert={{\kappa }_{s}}\left(d-{{d}_{0}} \right)$, for $d\leqslant {{d}_{0}}$ and zero for $d&gt;{{d}_{0}}$, where d is the distance between microtubules, ${{d}_{0}}$ is a measure of microtubule separation and ${{\kappa }_{s}}$ is a stiffness constant characterizing the strength of the steric force. The stiffness constant was chosen to be large enough that steric interactions dominate over thermal fluctuations but small enough to be comparable to forces produced by crosslinking motors. Hence, there is only a small probability that two microtubules will cross in the 2D  +  S model, dependent on their crossing angle and the forces acting upon them. In the 3D  +  S model the *Z*-dimension is equal to $2{{d}_{0}}$, allowing two layers of microtubules to lie close to and parallel with the confining walls and a central microtubule layer to lie a distance ${{d}_{0}}$ from either wall.

Key parameters of the model were based on previously measured values (supplementary table 1 and [13] (stacks.iop.org/PhysBio/16/046004/mmedia)). To reduce computation time the microtubule growth and motor speeds were chosen to be ten times faster as compared to a previous model that reproduced both contractile and extensile states [13], since the ratio of microtubule growth speed to motor speed was identified as a control parameter of network organization, within a reasonable range [13]. Microtubule numbers, *N*_MT_, reported as a packing fraction refer to the 2D  +  S model. The packing fraction, Φ, is defined as the total area occupied by the steady-state microtubule population divided by the total area of the simulation space: Φ  =  2*L*  ×  steric radius  ×  *N*_MT_/(16*L*)^2^. Packing fractions from 10%–100% were explored. Systems were simulated for at least 5 times as long as the average lifetime of the microtubules in order to reach a steady-state network organization, indicated by the steadiness of several large-scale metrics (see below).

All simulation configuration files and documentation for the data presented here can be found online at https://doi.org/10.17632/r9y953dd26.1.

### Crosslink classification

**Figure 2.**
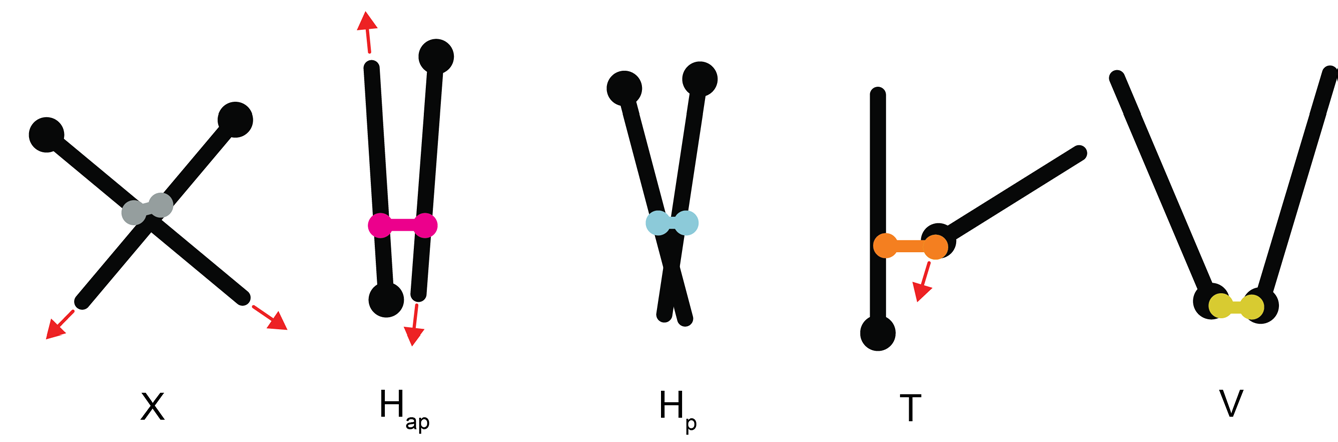
Classification of five different types of crosslinks. Red arrows indicate direction of relative microtubule motion.

To characterize the organization reached by the simulated systems, we analyzed the different types of connections that motors can make between microtubules (figure 2). We defined three types of connections between microtubule sides depending on the angle described by the microtubules, which is defined in [0, 𝜋] since microtubules are polar; *H*_p_ links connect the sides of ‘parallel’ microtubules at an angle 0  ⩽  *θ*  ⩽  𝜋/3, *H*_ap_ links connect the sides of ‘anti-parallel’ microtubules at an angle 2𝜋/3  <  *θ*  ⩽  𝜋 and *X* links connect microtubule sides at an angle 𝜋/3  <  *θ*  ⩽  2𝜋/3. We also defined two additional categories of connections for motors that had reached the microtubule plus-ends; *T* links connect the side of a microtubule to a microtubule plus-end and *V* links connect two plus-ends. The different link types have different effects on the microtubules they connect. Both *X* and *H*_ap_ links induce relative microtubule motion whereby each microtubule moves in the direction of its minus-end. However, while *H*_ap_ links slide microtubules along the same axis, microtubule motion due to *X* links is uncorrelated. *H*_p_ links induce negligible motion but bundle microtubules while processing along their sides. *T* links bring microtubule plus-ends together, while *V* links are static and maintain plus-end connections.

### Cluster analysis

To characterize the topologies of different networks we looked at the kinetics of microtubule cluster formation. Two microtubules belong to the same cluster if they are connected by any type of link or if they can be indirectly connected in this way via other microtubules. Similarly, two microtubules belong to the same ‘*V*-linked cluster’ if they are connected via a *V*-link or if they can be connected in this way via other microtubules. The size of the largest cluster and the largest *V*-linked cluster were monitored. *V*-linked clusters were further analyzed for calculation of the parameter *S*_v_, described below.

### Nematic order parameter

We used the usual 2D nematic order parameter, *S*, to quantitatively compare different models and conditions. For the 3D  +  S model, we disregarded the typically small *Z*-component of the microtubule’s direction vector and analyzed it as a 2D object. For a system with *N* particles *S* is defined as $S=\left\langle {\rm cos}2\left({{\theta }_{i}}-\widehat{\theta } \right) \right\rangle $ where ${{\theta }_{i}}$ is the direction of the *i*th particle and $\widehat{\theta }$ is the nematic director defined as the ensemble average, $\left\langle \ldots \right\rangle $, of the direction of the *N* particles [44]. In a completely random and isotropic system *S*  =  0 and for a perfectly aligned system *S*  =  1. We used a sampling window of size 2*L*  ×  2*L* in the *X*- and *Y*-directions in order capture local nematic order with a value of *N* large enough to avoid excessive noise. The sampling windows overlapped with a shift of *L*/4. S was calculated for each sampling window and then averaged over the entire simulation space. A single time-point was taken from the steady-state network and analyzed.

We also used a derived parameter, *S*_v_, to capture the degree of isotropy of asters in a network i.e. *S*_v_  =  0 indicates perfectly radial asters. *S*_v_ was calculated for a single time-point by selecting all *V*-linked clusters above a minimum size (10 microtubules), calculating S for each of these clusters and averaging the result. In calculations of *S*_v_ five time-points from the steady-state network were analyzed and averaged.

### Projected velocity

To quantify motor activity in our simulations the parameter $\left\langle \boldsymbol{v}\cdot \widehat{\boldsymbol{p}} \right\rangle $ was used, capturing the speed of microtubule motion along its axis. The microtubule velocity, $\boldsymbol{v}$, was calculated as $\boldsymbol{v}=\left[ \boldsymbol{x}\left(t+\tau \right)-~\boldsymbol{x}\left(t \right) \right]/\tau $ where $\boldsymbol{x}\left(t \right)$ is the position of the microtubule minus-end at time $t$. The microtubule’s unit direction vector $\widehat{\boldsymbol{p}}$ points along the microtubule’s axis towards the plus-end at time $t+\tau $. All microtubules present in frames $t$ and $t+\tau $ were used in this calculation. In the 3D  +  S model both ***x*** and ***p* ** were 2D vectors obtained by projection onto the *X*-*Y* plane. The final 4% of simulated time was analyzed and averaged to capture only the steady-state network behavior. The velocity was measured from the microtubules’ minus-ends to avoid a trivial contribution from microtubule growth at their plus-ends. The major contribution to $\left\langle \boldsymbol{v}\cdot \widehat{\boldsymbol{p}} \right\rangle $ comes from crosslinking motors moving towards microtubule plus-ends, which slides them backwards in the direction of their minus-ends and results in negative values of $\left\langle \boldsymbol{v}\cdot \widehat{\boldsymbol{p}} \right\rangle $.

## Results

Recent experiments have shown that the same set of components can produce three different types of microtubule-motor networks: active nematic networks and locally or globally contractile networks [13]. We investigated how spatial dimensionality and the inclusion or omission of steric interactions between filaments affects the capacity of a model to generate these archetypal network states. We compared the results of a 2D model omitting steric interactions (2D), a two-dimensional model including steric interactions that prohibit microtubule crossings (2D  +  S) and a thin 3D model including steric interactions (3D  +  S). Network organization was classified using a small set of characteristic quantities; the conventional nematic order parameter, *S*, which characterizes filament orientation; a derived quantity accounting for network topology and clustering, *S*_v_, to identify the presence of asters in a network and the numbers of different types of crosslinking motors (figure 2) [13].

### Active nematic states require steric interactions

**Figure 3.**
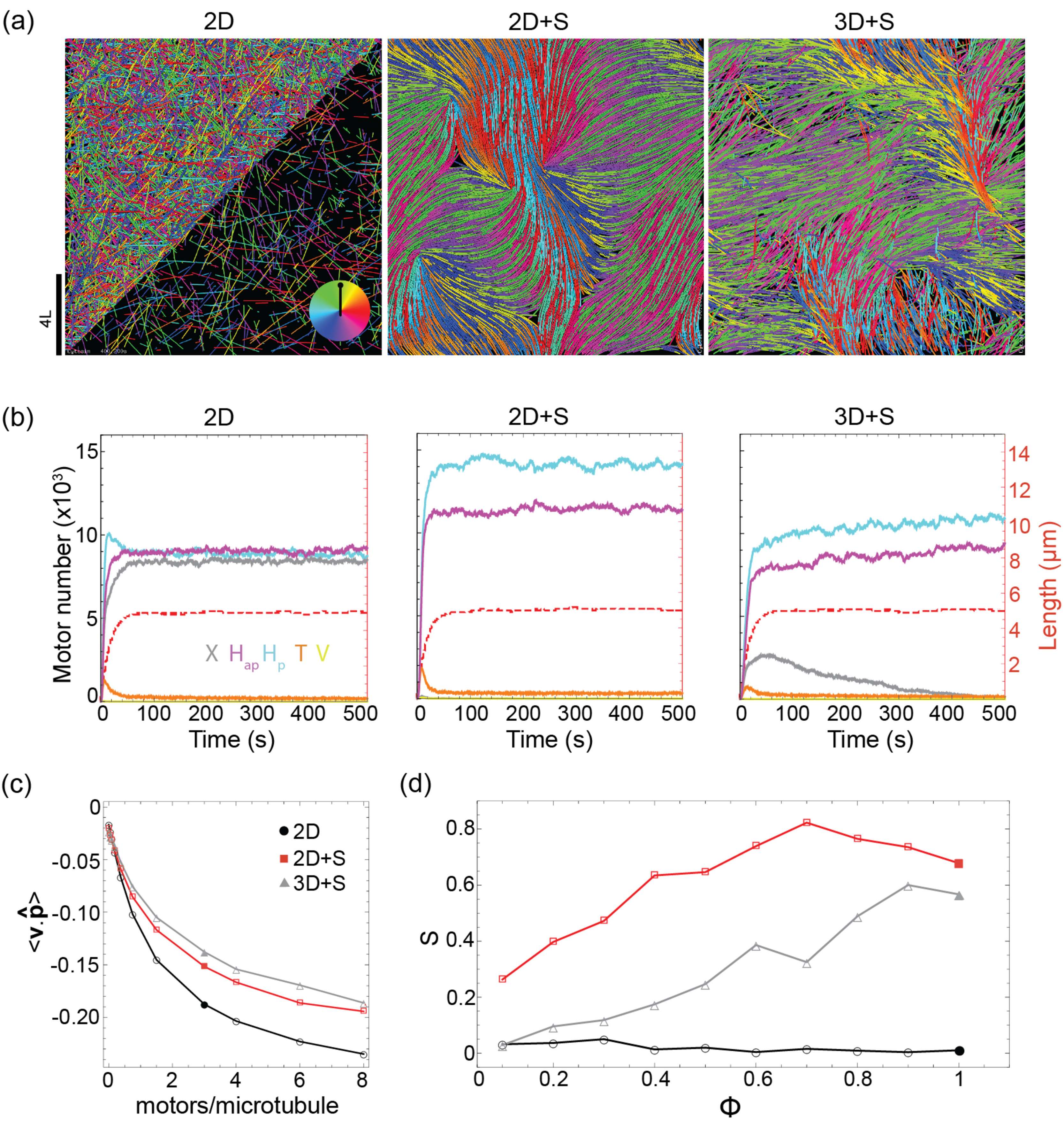
Exploration of active nematic network states. (a) Snapshots of steady-state networks generated in simulations of the 2D, 2D  +  S and 3D  +  S model. In all images the 3D simulation snapshot is a projection onto the *X*-*Y* plane. Microtubules are colored according to their orientation (see color wheel inset in left-hand image) and motors are not shown. In the bottom half of the left-hand image only a fraction of the microtubules is displayed for visual clarity. Packing fraction  =  100%, *N*_MT_  =  12 800, three motors/microtubule, *v*_*m*_/*v*_*g*_  =  1. For all other parameters see supplementary table 1. (b) Three kinetic plots characterizing the simulations represented in (a). Left-hand axis and solid lines show the numbers of the different link types over time (*X*: gray, *H*_ap_: purple, *H*_p_: blue, *T*: orange, *V*: yellow). Right-hand axis and dashed, red lines show the average microtubule length. (c) Average projected microtubule velocity $\left\langle \boldsymbol{v}\cdot \widehat{\boldsymbol{p}} \right\rangle $, in *µ*m/s, at varying microtubule packing fraction, $\phi $, for each model. (d) 2D nematic order parameter *S* at varying microtubule number for each model (2D: black circles, 2D  +  S: red squares, 3D  +  S: gray triangles). All other parameters as in (a). Solid data-points represent the simulations shown in (a).

We first investigated the capacity of our three models to form active nematic networks. This network state is favored by high densities of microtubules and a motor speed comparable to or faster than the microtubule growth speed [13]. In this regime motors cannot effectively reach microtubule plus-ends and dwell mostly on the microtubules’ sides. With the same simulation parameters defining microtubule and motor properties (supplementary table 1), we observed differences in the networks generated by each model, as illustrated by snapshots of the steady-state network organization (figure 3(a)).

Active nematic networks formed for both the 2D  +  S and 3D  +  S models that included steric interactions (figure 3(a) middle & right). However, in the 2D model lacking steric interactions the network was disorganized (figure 3(a) left). In the 2D model microtubules are free to cross and at steady-state the three types of side-side links (*X*, *H*_p_, *H*_ap_) (figure 2) dominated and were present in equal numbers (figure 3(b) left). Since these link types were defined by partitioning the range of possible connection angles in three equal parts, this is indicative of an isotropic network. While crosslinking motors acting on an isolated pair of microtubules can promote their alignment [30], this is generally not the case in dense networks where each microtubule is connected at multiple positions along its length. Instead, microtubules appear to ‘glide’ over other microtubules (supplementary figure 1(b)) and within periodic boundaries this did not induce nematic ordering on the time-scale of our simulations (supplementary figure 1(a) top).

In the 2D  +  S model microtubule crossings were penalized by steric interactions and the population of *X* links was negligible throughout the simulation (figure 3(b) middle). Microtubules began to align as soon as they nucleated, forming small bundles with local nematic order which fused and became larger as the microtubules grew to their steady-state length (supplementary Movie 1, supplementary figure 1(a) middle). The bundles were always of mixed-polarity, reflected in almost equal numbers of *H*_p_ and *H*_ap_ links (figure 3(b), middle). Motile topological defects were also observed in the network (figure 3(a) middle, supplementary figure 1(a) middle, supplementary Movie 1), as expected theoretically [18, 25, 26].

In the 3D  +  S model microtubules could cross in the *X*-*Y* plane while fulfilling steric constraints by moving passed one another in the *Z*-dimension. A significant population of *X* links formed at the crossings of unaligned microtubules at early times in the simulation when microtubules were short (figure 3(b) right, supplementary figure 1(a) bottom). However, as the microtubules grew longer steric interactions became more important and promoted alignment; local nematic order increased as *X* links were depleted in favor of *H* links. Eventually, a similar steady-state crosslink distribution as in the 2D  +  S model was established. Topological defects were also apparent (figure 3(a) right, supplementary figure 1(a) bottom, supplementary movie 1), but their boundaries were less distinct than in the 2D  +  S model due to microtubule crossings at the bundle ends.

*X* and *H*_ap_ links present in all three networks drove microtubule sliding. This activity can be captured by the parameter $\left\langle \boldsymbol{v}\cdot \widehat{\boldsymbol{p}} \right\rangle $ which is the component of a microtubule’s velocity directed along its axis. For the networks shown in figure 3(a) $\left\langle \boldsymbol{v}\cdot \widehat{\boldsymbol{p}} \right\rangle $ was negative (figure 3(c), solid points), because microtubules were slid backward by motors in the direction of their minus-ends (red arrows, figure 2). As the motor number per microtubule was increased from zero the magnitude of $\left\langle \boldsymbol{v}\cdot \widehat{\boldsymbol{p}} \right\rangle $ increased, showing that the activity is motor-dependent (figure 3(c)), as has been observed experimentally [16]. At low motor numbers per microtubule the sliding velocity increased approximately linearly and then saturated at higher motor numbers per microtubule as it approached the unloaded motor speed. Because *X* links are negligible in the 2D  +  S and 3D  +  S models in the steady-state (figure 3(b) middle & right), *H*_ap_ links alone drive bundle extension and locally correlated microtubule motion. Bundles continuously formed, extended and disintegrated as the network struggled to achieve a globally polarity-sorted state in a process of active turbulence characteristic of active nematic networks (supplementary movie 1) [16]. By contrast, both *H*_ap_ and *X* links were present in the 2D model (figure 3(b) left) and contributed equally to the sliding activity (figure 3(c)), resulting in isotropic, uncorrelated microtubule motion (supplementary movie 1).

To compare the propensity for nematic network formation in the different models, we tracked the microtubule density-driven transition from an isotropic to nematic state [45], with the 2D nematic order parameter, *S*. We observed that the 2D  +  S model had a higher degree of nematic order than the 3D  +  S model at all microtubule densities, while the 2D model was always isotropic (*S*  ≈  0) (figure 3(d)). This suggests that, at the microscopic scale of our model, the probability of microtubule crossings (in the *X*-*Y* plane) determines the degree of nematic order that a network can achieve, as previously observed [46]. The 2D  +  S model imposes the greatest restriction on crossings, with the combination of reduced spatial dimensionality and steric interactions, making it the most conducive model to nematic states. In contrast, in the 2D model where microtubules are free to cross, the nematic state is not achievable.

### Aster formation is hindered by steric interactions

Microtubule-motor systems form polar structures called asters under a wide variety of conditions, in particular if the motors are able to reach the end of the filaments and dwell there [11]; such networks are locally contractile. To investigate aster formation, we therefore set the microtubule growth speed to be six times slower than the motor speed (supplementary table 1), so that motors could effectively accumulate at microtubule plus-ends.

**Figure 4.**
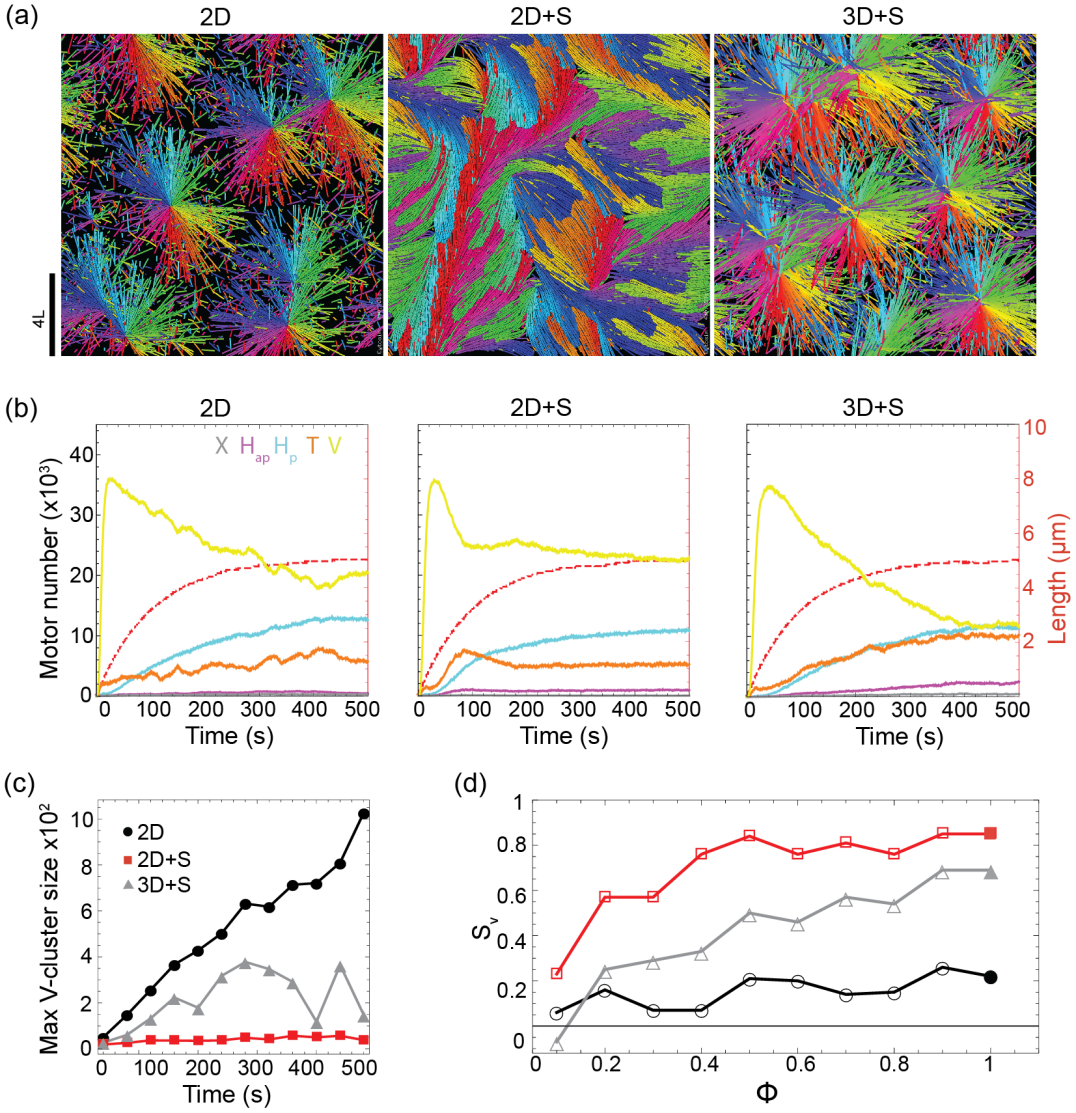
Exploration of aster states. (a) Snapshots of steady-state networks generated in simulations of the 2D, 2D  +  S and 3D  +  S model. Packing fraction  =  100%, *N*_MT_  =  12 800, 4 motors/microtubule, *v*_*m*_/*v*_*g*_  =  6. For all other parameters see supplementary table 1. (b) Three kinetic plots characterizing the simulations represented in (a). Left-hand axis and solid lines show the numbers of the different link types over time (*X*: gray, *H*_ap_: purple, *H*_p_: blue, *T*: orange, *V*: yellow). Right-hand axis and dashed, red lines show the average microtubule length. (c) Size of the maximum *V*-linked cluster over time from the simulations represented in (a). (d) Aster isotropy parameter, *S*_v_, at varying microtubule packing fraction, $\phi $, (2D: black circles, 2D  +  S: red squares, 3D  +  S: gray triangles). All other parameters as in (a). Solid data-points represent the simulations shown in (a).

At high microtubule densities we observed disparities between the three models. For the 2D and 3D  +  S models, which allow microtubule crossings, asters readily formed (figure 4(a), left & right). Radial arrays were achieved through the coincidence of microtubule plus-ends at the center of the aster and the overlapping of microtubules radiating from different asters. However, in the 2D  +  S model the microtubules did not form fully radial arrays but instead formed partially radial arrays (resembling fans), due to lack of space (figure 4(a) middle).

The observed differences were not reflected in the numbers of different link types, which appeared similar across the three models (figure 4(b)). The *V* link population peaked at the start of the simulation when the microtubules were short and was depleted as the average microtubule length increased allowing for more binding to the microtubules’ sides. In the steady-state *H*_p_ links were present in significant numbers, binding to the polarity-sorted arrays generated and maintained by the *T* and *V* links which, in sum, dominated the network.

To characterize the disparities between models we therefore looked at network topology and tracked the size of the largest *V*-linked cluster over time (figure 4(c)). In the 2D and 3D  +  S models, microtubules from different asters could cross and become crosslinked by motors, resulting in aster fusion (supplementary figure 2 top and bottom, supplementary movie 2). In the 2D model there are no restrictions on microtubule crossings or overlaps and therefore no limit to the maximum aster size; growth of the biggest *V*-linked cluster continued throughout the simulation. In the 3D  +  S model, steric interactions and the simulation volume imposed an upper limit on the number of microtubules that could be part of the same *V*-linked cluster. In the 2D  +  S model, where crossings and overlaps were prohibited, the maximum *V*-linked cluster size was severely limited and fusion did not occur (figure 4(c)).

To further determine how restricting microtubule crossings affected the formation of asters, we calculated an aster isotropy order parameter, *S*_v_, over a range of microtubule densities (figure 4(d)). In the 2D model where crossings are unrestricted, *S*_v_ was always low (≈0.2) indicating aster isotropy. In the 2D  +  S model radial arrays were observed at low microtubule densities but were replaced with the fan-like structures as the density increased, indicated by high values of *S*_v_. In the 3D  +  S model, aster formation was observed over a broad range of microtubule densities but *S*_v_ values were systematically lower than in the 2D model. Taken together these observations suggest that steric interactions between microtubules inhibit aster formation, restricting their size and isotropy and that this effect is most pronounced at high microtubule densities.

### Globally contractile states require *X* links to form long-distance connections across the network

**Figure 5.**
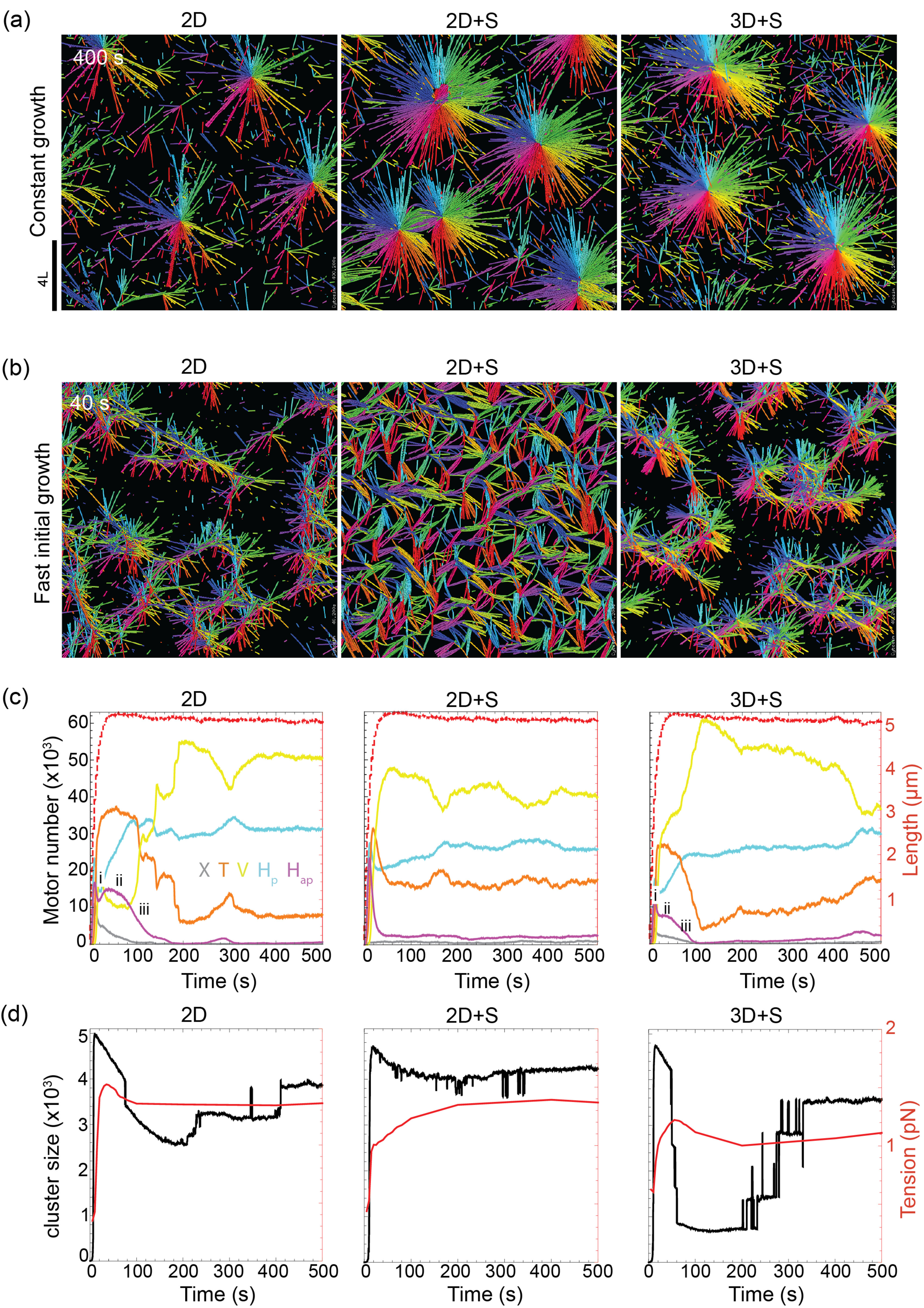
Exploration of globally contractile states. (a) Snapshots of steady-state networks generated in simulations of the 2D, 2D  +  S and 3D  +  S model under constant growth conditions. Packing fraction  =  40%, *N*_MT_  =  5120, 20 motors/microtubule, *v*_*m*_/*v*_*g*_  =  6. For all other parameters see supplementary table 1. (b) Snapshots of early network formation under ‘fast initial growth’ conditions (see text). All other parameters as in (a). (c) Three kinetic plots characterizing the simulations represented in (b). Left-hand axis and solid lines show the numbers of link types over time (*X*: gray, *H*_ap_: purple, *H*_p_: blue, *T*: orange, *V*: yellow). The multi-step contraction process is indicated by roman numerals. Right-hand axis and dashed, red lines show the average microtubule length. (d) Three kinetics plots characterizing the simulations represented in (b). The size of the maximum cluster is shown by the black curve. The average tension in the crosslinking motors is shown by the red curve.

Lowering the microtubule density and increasing the motor/microtubule ratio, we observed that all three models produced radial asters (figure 5(a)) with similar dynamics (supplementary figure 3(a), supplementary movie S3). However, a further disparity between models became evident by changing the microtubule growth conditions. Instead of constant growth speed, we modeled the physiological situation where microtubule growth speed correlates with effective tubulin concentration, which is initially high and drops as the microtubule mass increases.

In this ‘fast initial growth’ regime we observed an early transient, globally contractile state in the 3D  +  S and 2D model (figure 5(b)). At the start of the simulation microtubules grew quickly and reached their steady-state length before motors could crosslink and polarity-sort them (supplementary figure 3(b)), resulting in an initially isotropic network with many filament crossings (supplementary figure 3(c), supplementary movie 4). Motors subsequently bound to these crossings, forming *X* links, at which point the network was globally connected. The network then fractured in a multi-step contraction process: (i) Initially, there was fast, local polarity-sorting indicated by a small drop in *H*_ap_ links (figure 5(c) right & left). Here the isotropic network reorganized into a network of connected foci (figure 5(b), right & left). (ii) The network then underwent a transient period of global contraction as tension was transmitted between foci, which pulled on each other in all directions. During this period further polarity-sorting was frustrated, indicated by the plateau in the number of *H*_ap_ links (figure 5(c) right and left). During this stage the motors were under the highest tension (figure 5(d) right and left). (iii) The network finally ruptured into several small clusters (figure 5(d) right & left), and the disappearance of *H*_ap_ links during this stage marked the completion of polarity-sorting.

In the 3D  +  S and 2D models the initial population of *X* links form the interconnections between foci which drive the global contraction, keeping the network connected during phases (i) and (ii). Although in the 2D  +  S model fast initial growth also resulted in a fully connected network at early times (figure 5(d) middle), the absence of *X* links (figure 5(c) middle) meant that tension was not transmitted across the network. Polarity-sorting was always local and could be completed quickly; seen by the fast depletion of *H*_ap_ links (figure 5(c) middle). These results suggest that globally contractile states require that microtubules crossings and *X* links are present in the network.

## Discussion

We have investigated the effects of dimensionality and steric interactions on the organizational state space of networks of dynamic microtubules and end-dwelling motors, focusing on three archetypal network states; active nematic networks, asters and globally contractile networks. This state space was traversed by tuning recently identified control parameters of self-organization [13].

We found that microtubule crossings play important and distinct roles in each network state and that steric interactions and spatial dimensionality have a combined effect on microtubule crossing probability. Without steric interactions, the 2D model conveniently captured the network states in which microtubule crossings are critical. This model is therefore a good choice for representing the networks of large asters observed to fuse in some experimental systems [8–10]. However, it does not provide an upper limit to the number of microtubule plus-ends which can be gathered in one cluster and would contract beyond the maximum density reached experimentally [12]. Globally contractile networks were also well reproduced in this model. We observed that *X* links formed at the crossings of long microtubules were necessary to transmit tension between locally contracted foci and keep the entire network connected as the foci pulled on each other; a mechanism of global network contraction that has been described experimentally [12, 14]. The omission of steric interactions in 2D [47, 48] and 3D [49] microscopic models of crosslinked actin networks emphasizes the validity of this approach for studying contractile networks. On the other hand, the 2D model could not represent nematic network states which require alignment to dominate over crossings.

Conversely the 2D  +  S model readily formed nematic states; a high degree of nematic ordering was achieved over a broad range of microtubule densities and well-defined, motile topological defects were observed, as expected theoretically [18, 25, 26]. This model well reproduces the active liquid crystal behavior seen in experiments where microtubule bundles were confined to a single plane [17, 20, 21]. It has also been observed that microtubule bundling can be modulated by tuning steric interactions in 2D networks [50]. Asters could form in the 2D  +  S model, but only at low microtubule densities and they were limited in size. Furthermore, a globally contractile state could not be achieved since *X* links do not form in our 2D  +  S model.

**Figure 6.**
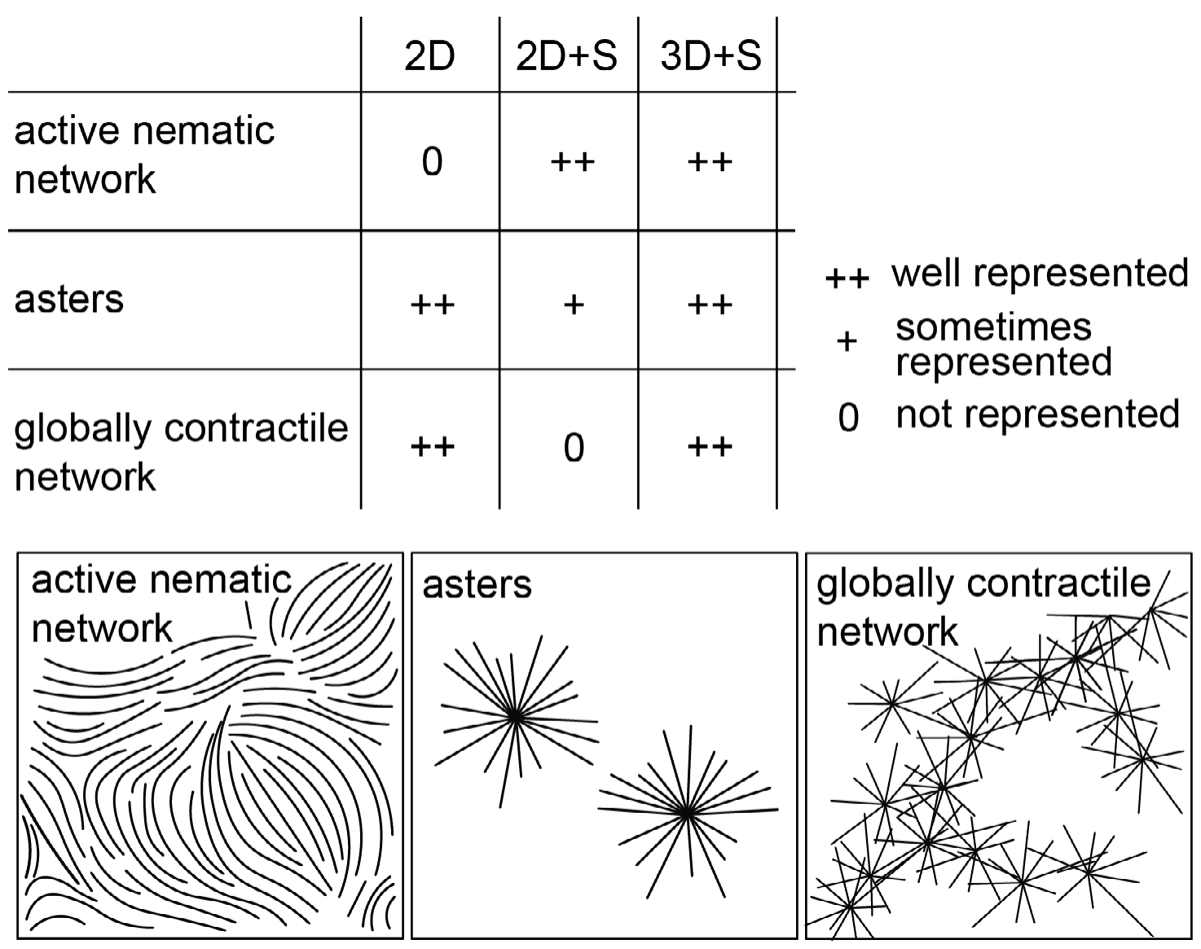
Schematic summarizing the state space of microtubule-motor self-organization represented by the three models.

Our results can be simply interpreted in light of the fact that crossings are unrestricted in our 2D model and highly restricted in our 2D  +  S model with strong steric interactions. In this context the 2D and 2D  +  S model are limiting cases, having opposite tendencies with respect to which network states they can produce (figure 6). The 3D  +  S model offers a good compromise as it includes non-overlap conditions in a geometry that does not preclude motor-mediated connections between the sides of unaligned microtubules (*X* links). The 3D  +  S model is thus the most versatile, allowing for the formation of contractile states and active nematics. Moreover, it has the additional potential for replicating unique behaviors of thin-3D active gels [13, 16, 17], that are not observable in a 2D model.

A practical consideration is that the implementation of a 3D model in computer code requires much greater effort than is required for a 2D model; code sharing and reuse is a solution to this challenge. Furthermore, computational cost is typically greater in three dimensions than in two and this can limit the practical value of a model. However, it is critical to seek physical accuracy when aiming to identify microscopic system properties that drive active networks between multiple states [13, 29, 41]. Neither our 2D nor our 2D  +  S model could capture the full variety of network states observed in experiments [13]. But pleasingly, our thin 3D  +  S model reproduced all archetypal network states for an extra computational time cost of only  ≈50%. The simulations presented in this study with 12 800 microtubules and 38 400 motors were calculated with one CPU core in 24 h for 2D models and in 38 h for the 3D model.

Comparing the state spaces of microtubule-motor self-organization represented by the three different models (figure 6), provides insight into the mechanisms driving the formation of three archetypal networks observed *in vitro*, particularly elucidating the role of microtubule crossings. Our results may inspire a modeler’s choices with regards to spatial dimensionality and steric interactions, bearing in mind the type of network under study (figure 6). With the exception of active gel monolayers [17, 20, 21], which would best be represented by a 2D  +  S model, our 3D model will ultimately provide the most realistic representation of most experimental systems. However, it may not always be the most appropriate choice given the additional computational cost, when a 2D model would suffice. Moreover, it may be possible to use an effective 2D interaction to penalize filaments crossing without forbidding them, in a 2D model representing a 3D system [39, 46], but we have not explored this possibility here. 3D  +  S models have so far been limited to thin volumes [13, 41] or low microtubule density regimes [28]. Exploring thick, dense systems remains an exciting goal that appears achievable in the near future.
